# Canagliflozin combined with metformin versus metformin monotherapy for endocrine and metabolic profiles in overweight and obese women with polycystic ovary syndrome: A single-center, open-labeled prospective randomized controlled trial

**DOI:** 10.3389/fendo.2022.1003238

**Published:** 2022-09-06

**Authors:** Jiaqi Zhang, Chuan Xing, Xiangyi Cheng, Bing He

**Affiliations:** Department of Endocrinology, Shengjing Hospital of China Medical University, Shenyang, China

**Keywords:** sodium-glucose co-transporter 2 inhibitors, canagliflozin, metformin, weight-loss, polycystic ovary syndrome

## Abstract

**Objectives:**

Canagliflozin (CANA), a kind of sodium-glucose cotransporter-2 (SGLT-2) inhibition, study in which the role of CANA monotherapy in polycystic ovary syndrome (PCOS) has been investigated, and it could become a novel option in the PCOS treatment. Nevertheless, trials focused on SGLT-2 combination therapy’s efficacy, and safety in PCOS patients are limited. This randomized controlled trial compared the efficacy and safety of CANA and metformin (MET) combination therapy and MET monotherapy in endocrine and metabolic profiles of overweight and obese women with polycystic ovary syndrome (PCOS).

**Methods:**

Fifty-one overweight or obese non-diabetic PCOS women between 18 and 40 years old were enrolled. Patients were randomly allocated to receive either CANA/MET or MET treatment. The CANA/MET group received CANA 100 mg once daily plus MET 1000 mg twice daily, while the MET group received MET 1000 mg twice daily for three months. Changes in menstrual pattern, anthropometric parameters, gonadal parameters, glucose and lipid homeostasis, and adverse events (AEs) were evaluated.

**Results:**

Compared with the MET group, women have a significantly lower level of total testosterone (TT), area under the curve for glucose (AUCGlu), and area under the curve for insulin (AUCIns) to AUCGlu ratio in the combination group. There were no significant differences in menstrual frequency, body weight, body mass index, follicle-stimulating hormone, luteinizing hormone, free androgen index, sex hormone-binding globulin, androstenedione, fasting blood glucose, fasting insulin, AUCIns, homeostasis model assessment-insulin resistance (HOMA-IR), triglycerides, total cholesterol, low-density lipoprotein cholesterol, apolipoprotein A1 (Apo A1), apolipoprotein B (Apo B), and APO B/A1 ratio. AEs were seen in 57.70% (15/26) and 68.00% (17/25) of patients in the CANA/MET and MET groups, respectively.

**Conclusions:**

In overweight and obese women with PCOS, CANA and MET combination therapy may be similar to MET monotherapy in improving menstrual frequency, weight control, hyperandrogenemia, and relieving insulin resistance. CANA/MET may have more benefits in reducing TT, AUCGlu, and the AUCIns/AUCGlu ratio within three months than MET monotherapy.

**Trial registration:**

ClinicalTrials.gov, NCT04973891.

## Introduction

According to different diagnostic criteria, polycystic ovary syndrome (PCOS) is one of the most prevalent reproductive endocrine disorders. It affects 4–21% of women of reproductive age (1, 2). On ultrasonography, hyperandrogenism (HA), ovulatory dysfunction, and polycystic ovaries are features of this syndrome (3, 4). In addition to these diagnostic features, obesity and insulin resistance (IR) are common abnormalities associated with PCOS (5). Approximately 50% of women with PCOS are overweight or obese (6), which could significantly amplify and worsen metabolic and reproductive outcomes regardless of PCOS phenotypes (7, 8). Hyperinsulinemia caused by IR is believed to promote HA in PCOS because insulin may augment luteinizing hormone (LH)-induced androgen production and reduce the liver’s sex hormone-binding globulin (SHBG) synthesis (9, 10). Excess androgen in PCOS women could aggravate IR and lead to compensatory hyperinsulinism, further enhancing ovarian theca cell androgen secretion (11–14). Overall, obesity, IR, and HA may interact with and influence one another, contributing to PCOS development. Besides, regardless of ovulatory status, women with PCOS still risk their fertility potential being reduced (15). This may be caused by, for example, pregnancy complications (16) and the alternations in oocyte competence (17) and in endometrial competence (18). Furthermore, it is suggested that these disorders may be exacerbated by obesity, HA, and IR *via* various mechanisms, such as the affection of the physiological microenvironment in the follicular fluid, inflammation, and oxidative damage (17, 18). Currently, there is no specific remedy or cure for PCOS (19), and the therapy for PCOS administration has typically focused on the control of symptoms (20).

Metformin (MET), the most extensively used insulin-lowering drug in PCOS (21), reduces hepatic glucose production, inhibits gluconeogenesis and lipogenesis, and enhances insulin sensitivity in peripheral tissues (22). Various MET functions in PCOS have been proposed, such as weight reduction, decreased serum testosterone levels, lipid metabolism disorder amelioration, and endothelial function improvement (23). It is well documented that obesity treatment is essential for PCOS management. A mere 5% reduction (24) in body weight could reduce IR, hyperinsulinemia, and HA; increase SHBG production, and improve abnormal reproductive measures (24, 25). For weight loss, it is found that MET monotherapy can achieve sound effects but is not perfect in morbidly obese PCOS women (26).

Sodium-glucose cotransporter-2 (SGLT-2) inhibitors, novel hypoglycemic oral drugs that promote renal glucose loss (27), are widely used clinically in patients with diabetes. Many studies have shown that SGLT-2 inhibitors can reduce fat mass (28), and blood pressure (28), ameliorate glucose homeostasis (29), alleviate oxidative damage and inflammation (30), and protect the cardiovascular system (31). In addition, several studies have substantiated the view that SGLT-2 inhibitors can significantly reduce weight in non-diabetic overweight and obese individuals with few adverse events (AEs) (32–36). Based on the advantages of both anthropometric and metabolic profiles, the emergence of SGLT-2 inhibitors for PCOS treatment has aroused general interest (27, 37). Cai et al. found that canagliflozin (CANA) was not inferior to MET in improving weight loss and IR, and its supplementation in PCOS patients should be considered (38). However, few trials have focused on SGLT-2 combination therapy’s efficacy and safety in PCOS patients.

Therefore, this randomized controlled trial (RCT) explored the difference in anthropometric indices, menstrual frequency, gonadal parameters, glucose and lipid homeostasis, and AEs between CANA/MET combination therapy and MET monotherapy in women with PCOS over three months. The present study aimed to provide additional options for PCOS treatment.

## Methods

### Participants

Patients in this open-label RCT were selected from the outpatient clinics of Shengjing Hospital of China Medical University Endocrinology Department, Shenyang, Liaoning, China, from April 2021 to March 2022.

### Ethics

This single-center, open-label, 1:1 RCT was examined and approved by the Scientific Research and New Technology Ethical Committee of the Shengjing Hospital of China Medical University (No.2021PS555K) and pre-registered at ClinicalTrials. gov (NCT04973891). All participants read and signed a written informed consent form before testing.

### Inclusion and exclusion criteria

Inclusion criteria: (i) 18-40 years (ii) Body mass index (BMI) ≥ 24 kg/m^2^ (iii) PCOS diagnosis fulfills the Rotterdam 2003 criteria phenotype B with HA and oligo-/anovulation (4) (iv) A negative serum pregnancy test before enrollment.

Exclusion criteria: (i) Patients who were pregnant, intended to become pregnant, were breastfeeding or did not agree to birth control. (ii) Medication history in the recent three months included oral contraceptive pills, SGLT-2 inhibitors, glucagon-like peptide-1 receptor agonists, thiazolidinediones, MET, and Chinese herbs. (iii) Comorbidities (diabetes, abnormal thyroid function-hyperthyroidism or hypothyroidism, 21-hydroxylase deficiency, hyperprolactinemia, androgen-secreting tumors, congenital adrenal hyperplasia, and Cushing syndrome), (all based on patient’ s medical records) (iv) Severe hepatic (alanine aminotransferase, aspartate aminotransferase > 3 times the normal value) or renal function (eGFR < 60 ml/min per 1.73 m2) damage. (v) Current or past (last three months) involvement in other interventional studies. (vi) 17α-dihydroxy-progesterone > 2ng/ml, (vii) Women with persistent or recurrent symptomatic urinary tract infection (UTI), gastrointestinal (GI) problems, or any other conditions that could endanger the patient’s safety.

### Study process

Eligible PCOS patients who provided consent were recruited and randomly allocated to either the CANA/MET group or the MET group. Randomization was performed using a computer-generated random number sequence. CANA and MET tablets were provided by Janssen Ortho, LLC, and Bristol-Myers Squibb Company, respectively. For CANA, subjects were required to take 100 mg once daily before breakfast; for MET, subjects were asked to take 1000 mg/day (500 mg twice daily with meals) for one week, with the dose increased to 2000 mg/day (1000 mg twice daily with meals), if tolerable. The management was for three months. All eligible patients were instructed to maintain their habitual diet, exercise level, and contraceptive use throughout the study period. They were also required to abstain from any drug with possible endocrine or metabolic effects.

Each participant completed assessments at two-time points: baseline and 12 weeks post-randomization. All PCOS subjects had to fast when measurements were taken. At the beginning of the study, data on body composition, glucose and lipid homeostasis, and sex steroid hormone concentration were measured and recorded. At the end of the study, the subjects underwent repeat assessments identical to the initial visit. We frequently contacted PCOS patients through weekly phone calls or communication tools, asking about their menstrual cycle and medication AEs, reminding them to take supplements daily, and arranging a convenient time for the next visit.

### Assessment of anthropometric indexes

Each subject’s weight and height were measured and recorded by a nurse to calculate body mass index [(BMI); weight (kg)/height (m2)] wearing light indoor clothing without shoes. We acknowledged that according to the WHO, overweight/obesity was defined as a BMI ≧ of 25 since the individuals included were all Chinese, and a BMI of 24 and 28 were cutoffs for overweight and obesity for both males and females over 18 years of age (39, 40). Height was measured using a standardized wall-mounted radiometer ( ± 0.1 cm) (Seca 71; Hamburg, Germany), and body weight was measured using a multi-frequency bioelectrical impedance analyzer (InBody 770 scanner; In-body Bldg; Seoul, Korea). Anthropometric indices were assessed at baseline and 12 weeks post-randomization.

### Assessment of menstruation

Due to all included PCOS patients meeting the Rotterdam 2003 criteria phenotype B (4), with ovulatory dysfunction (oligo-/anovulatory), irregular menstruation involved oligomenorrhea and amenorrhea. Oligomenorrhea refers to women with less than six menstrual periods within 12 months, while amenorrhea refers to individuals who have stopped menstruating for more than six months. Each bleeding counts as one menstrual cycle. Menstrual frequency recovery was defined as the recurrence of regular menstrual cycles in patients and was recorded at 12 weeks post-randomization.

### Assessment of biochemical parameters

Follicle-stimulating hormone (FSH) (mIU/mL) and LH (mIU/mL) levels were tested by chemiluminescent immunoassay. Total testosterone (TT) (ng/ml) was determined using an electrochemiluminescent immunoassay (ECLLA). HA was defined as TT was higher than 0.5ng/mL (17). Sex hormone-binding globulin (SHBG) (nmol/L) was tested using immunochemiluminescence (Unicel Dxl 800; Beckman Coulter, USA). Free androgen index (FAI) (%) was calculated as TT (nmol/L) × 100/SHBG (nmol/L) ratio, and TT (ng/mL) was converted to TT (nmol/L) divided by 3.467 (nmol/L). Androstenedione (AND) (ng/ml) was tested using luminescence. LH, FSH, TT, SHBG, FAI, and AND assessment was performed at baseline and 12 weeks post-randomization.

Glucose tolerance and insulin sensitivity were assessed at baseline and 12 weeks post-randomization using the oral glucose tolerance test (OGTT). Blood samples were taken at 0, 60, and 120 min after a sugar meal and analyzed for blood glucose (mmol/L) using the hexokinase-6 phosphate dehydrogenase method or the chemiluminescence (double-antibody sandwich) for blood insulin (μU/mL) (Abbott Architect ci 16200; Abbott, USA). The homeostasis model assessment of insulin resistance (HOMA-IR) was calculated as fasting insulin (FINS) (μU/mL) × fasting blood glucose (FBG) (mmol/L)/22.5. The area under the glucose curve (AUCGlu) (mmol/L · min) and insulin (AUCIns) (mU/L · min) were obtained by calculating the sum of the trapezoidal areas between 0, 60, and 120 min.

The deionization and enzyme method was used to evaluate triglyceride (TG) (mmol/L) concentration. Total cholesterol (TC) (mmol/L) was measured using the cholesterol oxidase method, and low-density lipoprotein cholesterol (LDL-C) (mmol/L) was measured using the chemically modified enzyme method. Apolipoprotein A1 (Apo A1) (g/L) and apolipoprotein B (Apo B) (g/L) were detected by immunoturbidimetry, and the Apo B/A1 ratio was also calculated (Abbott Architect ci 16200; Abbott, USA). The TG, TC, LDL-C, Apo A1, Apo B, and Apo B/A1 ratio assessment was performed at baseline and 12 weeks post-randomization. The AE severity was recorded and rated as mild, moderate, or severe.

### Sample size estimation

No evidence of PCOS in women treated with CANA/MET or MET combination has been reported. The sample size of the pilot study should be calculated based on its original purpose, comprehensive consideration, calculation, and analysis, so as to get the sample size. Based on the above characteristics, the explanation is as follows: The primary outcome was the three months change in body weight. The strategy of sample size calculation was based on the assumptions that the mean reduction of body weight (−2.10 ± 2.35) for the MET group, and the expectation of two more times reduction in the CANA/MET group (mean=−4.20), we required 21 subjects for each group. By considering α=0.05, power=80%, and an approximately 20% dropout rate, 50 patients were examined and equally assigned to each group (n=25).

### Statistical analysis

Continuous data were presented as mean, median, standard deviation (SD), and interquartile spacing; categorical data were presented as frequencies or percentages. AEs were calculated based on intention-to-treat principles, yet the treatment efficacy was measured using per-protocol analysis. First, normality was assessed using the D’Agostino and Pearson omnibus/Shapiro-Wilk test. The paired t-test or Wilcoxon signed-rank test was used for intragroup comparisons for continuous data. In contrast, an independent sample t-test or Mann-Whitney U test was performed for intergroup comparisons. For categorical variables, the chi-square test was used. Statistical significance was defined as a *P*-value < 0.05 (2-tailed). Results were obtained using GraphPad Prism Version 7.0 (GraphPad Software, Chicago, IL, USA) and SPSS (version 23.0; SPSS Inc., Chicago, IL, USA).

## Results

### Participants

**Figure 1:**
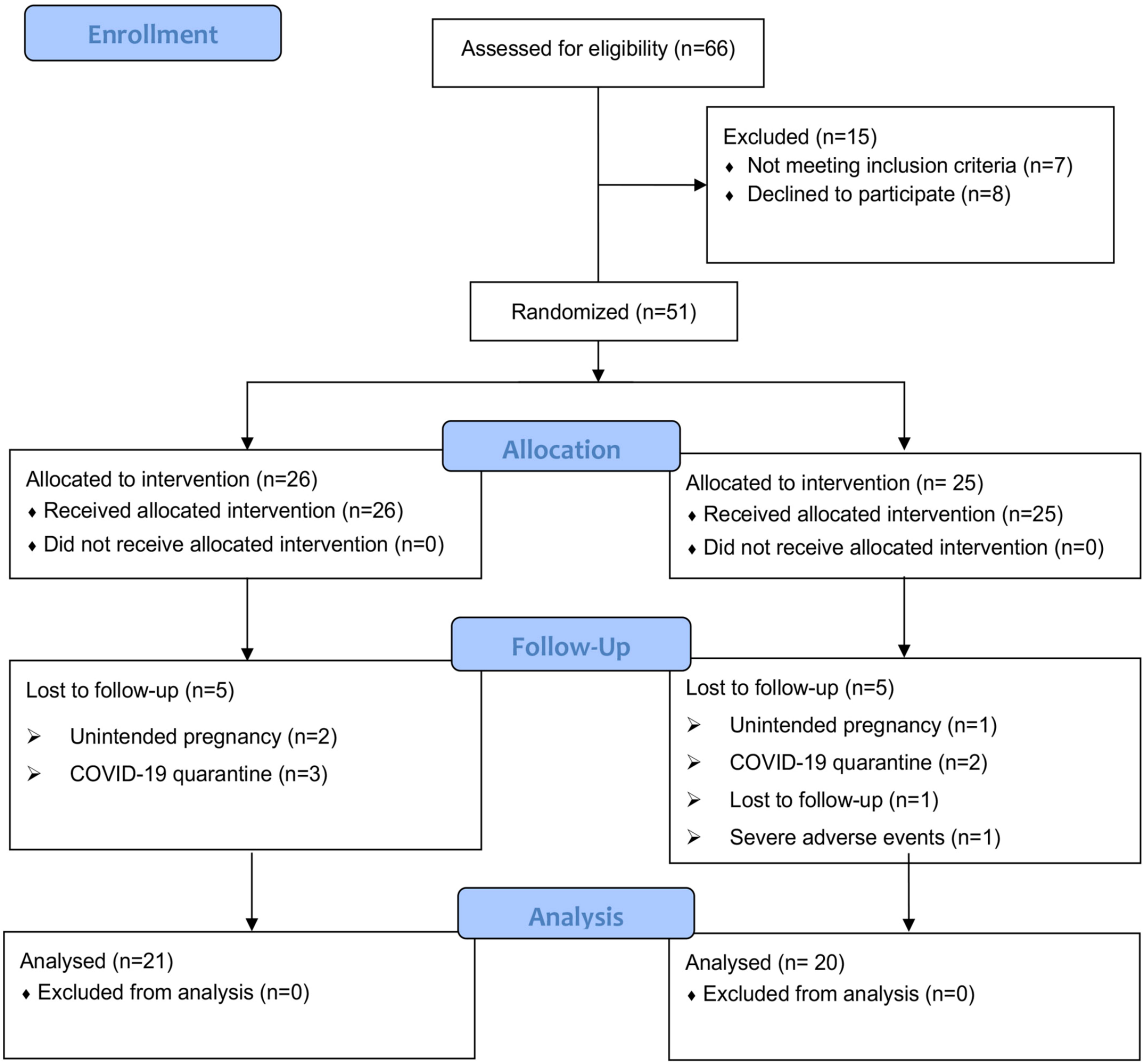
Patient selection flow diagram.

A total of 66 patients with PCOS based on the Rotterdam 2003 criteria were recruited from an outpatient endocrinology department. During the screening process, fifteen patients were excluded for definite reasons: four patients had a strong desire for pregnancy during the test; eight patients declined to participate; two patients had a history of taking multiple drugs (one with oral contraceptives and another with orlistat); one patient combined with other diseases (suffered from diabetes). Then, fifty-one PCOS patients who met the inclusion criteria were enrolled in the study, including 26 in the CANA/MET group and 25 in the MET group. Five patients dropped out in the CANA/MET group (2 patients with unintended pregnancy; 3 patients were affected by the COVID-19 quarantine). Five patients withdrew from the trial in the MET group (1 patient with unintended pregnancy; 2 were affected by the COVID-19 quarantine; 1 was lost to follow-up, and 1 had severe vaginal bleeding). Finally, 21 participants in the CANA/MET group and 20 in the MET group who completed the trial were included in the final analysis. Follow-up rates were 80.76% (21/26) and 80.00% (20/25), respectively (**Figure 1**).

### Baseline information

The two groups did not differ significantly in age, weight, or BMI (*P*=0.6118, *P* =0.2365, and *P* =0.1024, respectively). No statistically significant differences were found in interest outcomes according to baseline information. All baseline data were presented in **Table 1**.

**Table 1:** Demographic data and baseline characteristics of patients.

	CANA/MET (N = 21)	MET (N = 20)	*P* value
Age (years)	26.38 ± 5.89	25.55 ± 4.36	0.6118
Height (m)	1.62 ± 0.04	1.63 ± 0.05	0.4495
Body weight (kg)	81.23 ± 9.83	74.78 ± 8.91	0.2365
BMI (kg/m^2^)	31.11 ± 3.02	29.33 ± 3.19	0.1024
FSH (mIU/mL)	6.58 ± 1.54	6.05 ± 1.60	0.2800
LH (mIU/mL)	10.85 (6.36-16.22)	11.63 (9.69-16.87)	0.4304
TT (ng/mL)	0.95 (0.78-1.08)	0.89 (0.74-1.09)	0.7616
FAI (%)	28.62 ± 16.4	19.26 ± 9.46	0.0738
SHBG (nmol/L)	13.60 (8.55-20.15)	18.45 (13.13-21.98)	0.1626
AND (ng/ml)	3.57 ± 1.29	4.48 ± 1.42	0.0715
FBG (mmol/L)	5.70 (5.27-6.02)	5.30 (5.16-5.80)	0.1625
FINS (mU/L)	21.5 (14.35-24.20)	16.70 (14.58-24.33)	0.5919
AUCGlu (mmol/L*min)	1086 ± 208.7	985.3 ± 160.7	0.0915
AUCIns (mU/L*min)	14808 ± 6668	13867 ± 7201	0.6664
AUCIns/AUCGlu	13.97 ± 6.83	13.90 ± 6.55	0.9728
HOMA-IR	5.70 (3.38-6.08)	4.25 (3.26-6.44)	0.6515
TG (mmol/L)	1.54 (1.09-2.01)	1.49 (1.07-1.74)	0.6668
TC (mmol/L)	4.90 ± 0.93	4.74 ± 0.63	0.5353
LDL-C (mmol/L)	3.06 ± 0.97	3.01 ± 0.54	0.8401
Apo A1 (g/L)	1.16 ± 0.15	1.25 ± 0.20	0.1516
Apo B (g/L)	0.98 ± 0.26	0.88 ± 0.18	0.2032
Apo B/A1	0.85 ± 0.23	0.72 ± 0.18	0.0732

CANA, canagliflozin; MET, metformin; BMI, body mass index; FSH, follicle stimulating hormone; LH, luteinizing hormone; TT, total testosterone; FAI, free androgen index; SHBG, sex hormone-binding globulin; AND, androstenedione; FBG, fasting blood glucose; FINS, fasting insulin; AUCGlu, area under the glucose curve; AUCIns, area under the insulin curve; HOMA-IR, homeostasis model assessment-insulin resistance; TG, triglycerides; TC, total cholesterol; LDL-C, low-density lipoprotein cholesterol; Apo A1, Apolipoprotein A1; Apo B, Apolipoprotein B.

### Assessment of anthropometric parameters

No significant differences were found in body weight [CANA/MET: -6.66 ± 4.24 vs. MET: -5.85 ± 3.32; (*P*=0.5386)] and BMI [CANA/MET: -2.49 ± 1.55 vs. MET: -2.20 ± 1.30; (*P*=0.5441)] between the two groups. Within-group comparisons showed a significant decrease in body weight and BMI in the CANA/MET group (*P* < 0.0001 and *P* < 0.0001, respectively) and the MET group (*P* < 0.0001 and *P* < 0.0001, respectively) (**Table 2**).

**Table 2:** Information of 12-weeks post treatment and changes in endocrine and metabolic profile.

	CANA/MET (N = 21)		MET (N = 20)		*P* value (Change)
	12 weeks	Change from baseline	12 weeks	Change from baseline	
**Anthropometric characteristics**					
Body weight (kg)	75.40 ± 8.68*^d^*	-6.66 ± 4.24	72.49 ± 9.97*^d^*	-5.85 ± 3.32	0.5386
BMI (kg/m^2^)	28.62 ± 2.91*^d^*	-2.49 ± 1.55	27.14 ± 3.50^d^	-2.20 ± 1.30	0.5441
**Gonadal hormones**					
FSH (mIU/mL)	5.84 ± 2.24	-0.75 ± 2.51	5.36 ± 1.94	-0.68 ± 2.17	0.9309
LH (mIU/mL)	8.59 (3.96-12.16)	-1.91 (-7.40 to 2.49)	10.27 (8.22-13.61)	0.42 (-7.10 to 4.19)	0.1990
TT (ng/mL)	0.53 (0.45-0.84)*^d^*	-0.33 ± 0.23	0.71 (0.55-0.91)*^a^*	-0.18 ± 0.18	***0.0233***
FAI (%)	19.15 ± 13.19*^a^*	-9.47 ± 11.65	14.14 ± 12.57	-5.11 ± 7.40	0.1631
SHBG (nmol/L)	13.6 (9.55-24.10)	0.10(-3.45 to 5.30)	22.35(14.78-26.70)*^a^*	2.95 (-2.15-10.30)	0.4579
AND (ng/ml)	3.22 ± 1.35	-0.36 ± 1.17	3.79 ± 2.21	-0.39 ± 1.58	0.9555
**Glucose and lipid-related parameters**					
FBG (mmol/L)	5.20 (4.88-5.35)*^c^*	-0.33 (-0.95 to -0.05)	5.30 (4.96-5.42)	-0.11(-0.49 to 0.1)	0.1711
FINS (mU/L)	12.0 (8.20-20.15)*^c^*	-7 (-10.4 to -2)	14.70 (10.80-20.40)*^b^*	-4.2 (-9.8 to -0.7)	0.4565
AUCGlu (mmol/L*min)	928.3 ± 124.5*^b^*	-158 ± 225.4	988.5 ± 129.0	2.63 ± 180.7	***0.0182***
AUCIns (mU/L*min)	10543 ± 6888*^b^*	-4264 ± 5627	11691 ± 5212	-2640 ± 6108	0.3869
AUCIns/AUCGlu	11.12 ± 7.12*^a^*	-2.86 ± 5.71	11.76 ± 4.64*^a^*	0.51 ± 0.61	***0.0164***
HOMA-IR	3.14 (1.91-4.71)*^c^*	-1.83 (-3.01 to -0.96)	3.51 (2.36-4.71)*^b^*	-1.29 (-2.9 to -0.05)	0.4015
TG (mmol/L)	1.20 (0.84-1.63)*^a^*	-0.27 ± 0.51	1.43 (1.03-2.06)	-0.05 ± 0.59	0.2011
TC (mmol/L)	4.54 ± 0.80*^a^*	-0.22 ± 0.43	4.54 ± 0.52	-0.27 ± 0.48	0.7954
LDL-C (mmol/L)	2.83 ± 0.70	-0.12 ± 0.49	2.83 ± 0.49	-0.19 ± 0.50	0.6894
Apo A1 (g/L)	1.20 ± 0.21	-0.02 ± 0.33	1.25 ± 0.26	-0.02 ± 0.16	0.9465
Apo B (g/L)	0.92 ± 0.26	-0.05 ± 0.13	0.90 ± 0.13	0.01 ± 0.17	0.2887
Apo B/A1	0.78 ± 0.23*^a^*	-0.08 ± 0.14	0.74 ± 0.16	0.02 ± 0.22	0.1450

PCOS, polycystic ovary syndrome; CANA, canagliflozin; MET, metformin; BMI, body mass index; FSH, follicle-stimulating hormone; LH, luteinizing hormone; TT, total testosterone; FAI, free androgen index; SHBG sex hormone-binding globulin; AND, androstenedione; FBG, fasting blood glucose; FINS, fasting insulin; AUCGlu, area under the glucose curve; AUCIns, area under the insulin curve; HOMA-IR, homeostasis model assessment-insulin resistance; TG, triglycerides; TC, total cholesterol; LDL-C, low-density lipoprotein cholesterol; Apo A1, apolipoprotein A1; apo B, apolipoprotein B; N/A, not applicable.

**Bold** and italic fonts indicate statistically significant between the two groups.

^a^ P < 0.05, vs. baseline and 12-week visits.

^b^ P < 0.01, vs. baseline and 12-week visits.

^c^ P < 0.001, vs. baseline and 12-week visits.

^d^ P < 0.0001, vs. baseline and 12-week visits.

### Assessment of menstruation and gonadal parameters

After 12 weeks of treatment, an improvement in menstrual cycle irregularity was detected in CANA/MET group (80.95%, 17/21) and MET (80.00%; 16/20). There was no significant difference between the two interventions (*P*=0.6228). There was a clinically significant decrease in TT in the CANA/MET group compared to MET [CANA/MET: -2.49 ± 1.55 vs. MET: -2.20 ± 1.30; (*P*=0.0233)]. No differences were noted in FSH [CANA/MET: -0.75 ± 2.51 vs. MET: -0.68 ± 2.17; (*P*=0.9309)], LH [CANA/MET: -1.91(-7.40 to 2.49) vs. MET: 0.42(-7.10 to 4.19); (*P*=0.1990)], FAI [CANA/MET: -9.47 ± 11.65 vs. MET: -5.11 ± 7.40; (*P*=0.1631)], SHBG [CANA/MET: 0.10(-3.45 to 5.30) vs. MET: 2.95(-2.15-10.30); (*P*=0.4579)], and AND [CANA/MET: -0.36 ± 1.17 vs. MET: -0.39 ± 1.58; (*P*=0.9555)]. Within-group comparisons showed that both groups had significantly lower TT levels (*P* < 0.0001 and *P* = 0.0343, respectively). In the CANA/MET group, the FAI (*P* =0.0457) at 12 weeks decreased significantly compared to baseline, but no changes were observed in the MET group. In the MET group, SHBG (*P*=0.0303) at 12 weeks increased significantly compared to that at baseline, but no changes were observed in the CANA/MET group. Both groups showed no significant changes in FSH, LH, or AND levels after treatment (**Table 2**).

### Glucose homeostasis assessment

Participants in the CANA/MET group had a significant decrease in AUCGlu [CANA/MET: -158 ± 225.4 vs. MET: 2.63 ± 180.7; (*P*=0.0182)] and the AUCIns/AUCGlu ratio [CANA/MET: -2.86 ± 5.71 vs. MET: 0.51 ± 0.61; (*P*=0.0164)] compared with MET. There were no significant differences in FBG [CANA/MET: -0.33(-0.95 to -0.05) vs. MET: -0.11(-0.49 to 0.1); (*P*=0.1711)], FINS [CANA/MET: -7(-10.4 to -2) vs. MET: -4.2(-9.8 to -0.7); (*P*=0.4565)], AUCIns [CANA/MET: -4264 ± 5627 vs. MET: -2640 ± 6108; (*P*=0.3869)], and HOMA-IR [CANA/MET: -1.83(-3.01 to -0.96) vs. MET: -1.29(-2.9 to -0.05); (*P*=0.4015)]. Within-group comparisons revealed that both groups had significantly lower FINS levels (*P*=0.0003 and *P*=0.0041, respectively), the AUCIns/AUCGlu ratio (*P*=0.0327, and *P* =0.0255, respectively), and HOMA-IR (*P* =0.0002 and *P* =0.0028, respectively). Decreased FBG, AUCGlu, and AUCIns levels were observed in the CANA/MET group (*P*=0.0007, *P* =0.0044, and *P* =0.0024, respectively). However, these differences were not observed in the MET group (**Table 2**).

### Assessment of lipid homeostasis

No significant differences were found in all lipid parameters: TG [CANA/MET: -0.27 ± 0.51 vs. MET: -0.05 ± 0.59; (*P*=0.2011)], TC [CANA/MET: -0.22 ± 0.43 vs. MET: -0.27 ± 0.48; (*P*=0.7954)], LDL-C [CANA/MET: -0.12 ± 0.49 vs. MET: -0.19 ± 0.50; (*P*=0.6894)], Apo A1 [CANA/MET: -0.02 ± 0.33 vs. MET: -0.02 ± 0.16; (*P*=0.9465)], Apo B [CANA/MET: -0.05 ± 0.13 vs. MET: 0.01 ± 0.17; (*P*=0.2887)], and the Apo B/A1 ratio [CANA/MET: -0.08 ± 0.14 vs. MET: 0.02 ± 0.22; (*P*=0.1450)]. The TG and TC levels and the Apo B/A1 ratio declined from baseline only in the CANA/MET group (*P* =0.0314, *P*=0.0396, and *P* =0.0377, respectively). Both groups showed no significant changes in LDL-C, Apo A1, and ApoB levels after treatment (**Table 2**).

### AE assessment

AEs were seen in 57.70% (15/26) and 68.00% (17/25) patients in the CANA/MET and MET groups, respectively. Only one subject in the MET group had to withdraw due to severe vaginal bleeding. The details are summarized in **Table 3**.

**Table 3:** AEs of two treatment groups.

	CANA/MET (N = 26)	MET (N = 25)
Patients with AEs		
Severe AEs		
Vaginal bleeding	0	1
Mild and moderate AEs		
Nausea	11	14
Abdominal discomfort	1	4
Abdominal pain	2	1
Bloating	0	1
Diarrhea	4	8
Loss of appetite	2	4
Anorexia	1	0
Acid reflux	1	1
Headache	1	0
Dizziness	3	0
Asthenia	1	0
Bitter mouth	0	1

CANA, canagliflozin; MET, metformin; AEs, adverse events.

## Discussion

To our knowledge, this is the first 3-month randomized clinical trial comparing the efficacy and safety of CANA (100 mg once daily)/MET (1000 mg twice daily) and MET (1000 mg twice daily) in overweight and obese women with PCOS. Our results supported MET as conventional therapy for PCOS, given its amelioration of menstrual frequency, body weight, BMI, TT, FINS, HOMA-IR, and AUCIns/AUCGlu either combined with CANA or as monotherapy. In addition, we found that CANA/MET might be more beneficial in reducing TT, AUCGlu, and the AUCIns/AUCGlu ratio than MET monotherapy within three months. CANA/MET supplementation may be similar to MET monotherapy in PCOS administration to improve menstrual pattern, weight control, and HOMA-IR in overweight and obese PCOS women.

Weight loss is considered essential in PCOS management. In our study, participants in the CANA/MET group had a mean weight loss of 5.83 kg (7% of their body weight), and those in MET had 2.29 kg (3% of their body weight). However, the difference between the two groups did not reach statistical significance. This result agrees with an RCT that found that the effects of dapagliflozin (DAPA) (10 mg once daily)/MET (2000 mg once daily) combination therapy may be similar to those of MET (2000 mg once daily) monotherapy in promoting weight loss in PCOS patients (41). Furthermore, a similar non-significant difference in weight loss was reported in two RCTs that administered SGLT-2 inhibitors (empagliflozin 25 mg daily; canagliflozin 100 mg daily) monotherapy compared with MET in women with PCOS (38, 42). So far, the efficacy of SGLT-2 inhibition in weight control compared to MET in PCOS women has been rarely reported in the previous literature, with only above mentioned three RCTs. Interestingly, a meta-analysis involving seven studies with 2297 participants indicated that SGLT-2 inhibitor plus MET was superior to MET for weight control in people with type 2 diabetes for no more than 52 weeks (43). Another meta-analysis of eight studies with 750 individuals found that SGLT-2 inhibitor monotherapy for 12 weeks or more could lead to modest weight loss in non-diabetic overweight and obese individuals (44). This discordance might be due to the relatively short duration of the intervention or the different diseases and populations. Evidence related to the SGLT-2 combination strategy for PCOS treatment is rare. Further studies on the appropriate dosage and duration of SGLT-2 inhibitors are urgently required.

There was an improvement in menstrual cycle frequency in the CANA/MET and MET groups, with no significant difference. This result is in line with that of Cai et al. (38). For gonadal hormones, we found that the significant change in TT and FAI with both treatments from baseline was consistent with previous study findings, showing that DAPA/MET or MET could reduce TT and FAI (41). However, the results were inconsistent with some studies on SGLT-2 inhibitor monotherapy in PCOS (38) (42, 45). For instance, Tan et al. illustrated that supplementation with licogliflozin (50 mg three times daily) had no significant effect on reducing TT and FAI within two weeks compared to placebo (45). After 12 weeks of empagliflozin (EMPA) (25 mg once daily) intake, Javed et al. reported no significant decrease in TT and FAI (42). A recent study by Cai et al. also suggested that CANA (100mg once daily) had no significant effect on the reduction of TT and FAI at 12 weeks (38). Therefore, though in our trial we found that CANA/MET may be superior to MET in the reduction of TT in women with PCOS. The significant difference in FAI, an indicator that better discriminated PCOS than TT (46), did not exist between the two interventions. Therefore, we should be cautious not to draw definite conclusions due to the small sample size.

Metformin corrects endocrine and metabolic abnormalities in women with PCOS; it counteracts IR by inhibiting hepatic glucose production (22). By reducing the maximum kidney’s glucose reabsorptive capacity and glucosuria threshold, SGLT2 inhibitors enhance glucose excretion, reducing fasting and postprandial plasma glucose levels and improving insulin secretion and insulin sensitivity (47) Our study found that CANA/MET and MET could reduce FINS and HOMA-IR, with no significant difference among the treatment groups. Consistent with a recent randomized study by Tan et al., FINS and HOMA-IR levels declined significantly after two weeks of licogliflozin treatment (50 mg three times daily) in women with PCOS (45). Cai et al. also indicated that CANA was not inferior to MET regarding FINS and HOMA-IR reduction after 12 weeks of intervention (38). Javed et al. demonstrated similar results based on treatment with EMPA (25 mg once daily) for 12 weeks (42). Furthermore, a comparative 24-week study of patients with PCOS found that 10 mg DAPA daily or 10 mg of DAPA with 2,000 mg of MET daily significantly reduced patients’ HOMA-IR; however, the difference between the two interventions was not statistically significant (41). In our study, AUCGlu, AUCIns, and AUCIns/AUCGlu ratio decreased prominently in the CANA/MET group after 12 weeks; only AUCGlu and the AUCIns/AUCGlu ratio showed a statistically significant difference between the two comparisons. The AUCIns/AUCGlu ratio is relevant to pancreatic beta-cell dysfunction and the incidence of diabetes (48). Currently, there is only one related study examining AUCGlu and AUCIns between licogliflozin and placebo in PCOS (45), which found that licogliflozin (50 mg three times daily) could result in significant reductions in AUCGlu and AUCIns levels after a 2-week trial. No relevant studies have focused on the AUCIns/AUCGlu ratio.

In our study, the serum TG levels decreased significantly in the CANA/MET group. No significant differences were found in TG and LDL-C levels between the two treatments. SGLT-2 inhibition was found to affect the plasma lipid profile of diabetic patients by decreasing TG levels and increasing LDL-C levels (49). The LDL-C levels in the CANA/MET group were not altered from baseline, which is consistent with Cai et al. reports (38). Literature suggests that an increased Apo B/A1 ratio may worsen endocrine and metabolic profiles in women with PCOS. Also, it might be a valuable tool to screen PCOS intensity by evaluating IR and metabolic syndrome (50). We found that the Apo B/A1 ratio declined after CANA and MET combination therapy. Nevertheless, due to the small sample size and limited duration, trials with larger sample sizes are needed.

In our trial, CANA and MET were well tolerated by most patients. Only one patient withdrew owing to serious AEs and vaginal bleeding, which was considered unrelated to the study drug. Surprisingly, no UTI problems were observed in the CANA/MET therapy group. We speculate on possible reasons for the results. On the one hand, before starting medications, each participant was told to drink more water throughout the entire intervention period, also for the MET group. On the other hand, this may be because patients under this treatment had a small dose of CANA (100 mg q.d.) rather than CANA (300 mg q.d.). Nevertheless, we should still focus on the AEs after SGLT-2 inhibition supplementation, due to the small sample size. Most of the patients had mild or moderate GI problems in both groups, consistent with Cai et al. reports (38). Moreover, most AEs appeared in the initial stage of the experiment, especially after 1–3 weeks.

Our study has several strengths. Firstly, this is the first time assessing the differences between CANA/MET combination therapy and MET monotherapy in PCOS management. Until now, four clinical studies focusing on SGLT-2 inhibitors in PCOS management, and only one study compared the difference between DAPA/MET and MET monotherapy (41). The reason for selecting CANA as an intervention is that the primary outcome was set to be body weight, and CANA has displayed a better function of glucose excretion than DAPA. The second advantage is that the AUCIns/AUCGlu ratio after SGLT-2 inhibition supplementation in PCOS women has been assessed for the first time. AUCGlu and the AUCIns/AUCGlu ratio significantly lowered after combination therapy, and glucose metabolism amelioration is essential to PCOS management and its long-term complications. In our trial, we found that CANA/MET may be more beneficial in improving TT. It is suggested that SGLT-2 inhibition could lower total fat mass in PCOS rats with HA (28). Interestingly, though we did not focus on the change of fat mass, there was a significantly lower level of TG in the CANA/MET group compared with the baseline. In the MET group, no significant decrease in TG levels was seen. We speculate that SGLT-2 inhibition may reduce the effect of lipotoxicity, thereby ameliorating the metabolism of androgens in PCOS. Further basic and clinical studies are needed to investigate this issue. The present study has several limitations. First, this study was a single-center, open-label, lack-of-a-placebo controlled clinical design such that physicians and PCOS patients were not blinded to the medication. In the process of study design, we told all the included patients about their specific therapy strategies. The absence of blinding could make a possible subjective bias inevitable; this weakness, nevertheless, may be offset by the benefits of communicating with patients regularly, showing concern for patients, and responding to the questions as quickly as possible and thereby achieving better patient adherence as well as improving therapeutic relationships. Most of our patients were lost to follow-up for objective reasons like the COVID-19 pandemic. This could have impacted the final results’ reliability despite the rate being just 20%. Second, residual confounding factors, such as individual life modifications (dietary pattern and/or exercise), may affect the outcomes despite considering many potential confounders. Actually, though all eligible patients were instructed to maintain their habitual diet, exercise level, and contraceptive use throughout the study period, this was not monitored to ensure compliance. If the included patients did alter their habitual diet, exercise level, and contraceptive use, the results we achieved might not be due to interventions alone. There is a need for a specific diet, exercise, and contraceptive use monitoring in future studies. In addition, this trial had a relatively small sample size, and its period was too short to evaluate SGLT-2 inhibitor long-term effects in combination with MET in PCOS management. Finally, we focused only on gonadal and metabolic parameters between the two groups. Other indicators associated with body composition, blood pressure, and oxidative stress were not assessed.

SGLT-2 inhibitors may be promising new therapeutic drugs for PCOS. However, whether used alone or in combination with other agents, its efficacy still needs to be explored. It is essential to understand the mechanisms underlying SGLT-2 inhibition in PCOS and its long-term complications. The sample size should be expanded in further clinical trials, and a meta-analysis should be conducted to obtain high-quality conclusions. More studies are needed to be carried out to determine more efficient PCOS therapies.

## Conclusion

In overweight and obese women with PCOS, CANA and MET combination therapy may be similar to MET monotherapy in improving menstrual frequency, weight control, HA, and relieving IR. Compared with MET monotherapy, CANA/MET may have more benefits in reducing TT, AUCGlu, and AUCIns/AUCGlu ratio within three months. Additional trials are necessary to assess the SGLT-2 inhibitor supplementation’s long-term effects in patients with PCOS.

## Data availability statement

The raw data supporting the conclusions of this article will be made available by the authors, without undue reservation.

## Ethics statement

Ethical approval was obtained from the Scientific Research and New Technology Ethical Committee of the Shengjing Hospital of China Medical University (No.2021PS555K). Written informed consent was obtained from all participants prior to inclusion in the study. The patients/participants provided their written informed consent to participate in this study. Written informed consent was obtained from the individual(s) for the publication of any potentially identifiable images or data included in this article.

## Author contributions

JZ and BH conceived, designed, and performed the experiments; JZ, XC, and CX analyzed the data; JZ wrote the paper. BH reviewed and edited the final manuscript. All authors read and approved the final manuscript.

## Funding

This work was supported by a grant from the National Natural Science Foundation of China (grant no. 81570765), the Science and Technology Department people’s livelihood Science and Technology Joint Program Funding of Liaoning Province (No. 2021JH2/10300125), and the “345 Talent Project” of Shengjing Hospital of China Medical University.

## Acknowledgments

We want to thank all the patients and their families, without them this study could never have been completed. We wish them all the best.

## Conflict of interest

The authors declare that the research was conducted in the absence of any commercial or financial relationships that could be construed as a potential conflict of interest.

## Publisher’s note

All claims expressed in this article are solely those of the authors and do not necessarily represent those of their affiliated organizations, or those of the publisher, the editors and the reviewers. Any product that may be evaluated in this article, or claim that may be made by its manufacturer, is not guaranteed or endorsed by the publisher.

## Abbreviations

AND, androstenedione; Apo A1, apolipoprotein A1; Apo B, apolipoprotein B; AUCGlu, area under the glucose curve; AUCIns, area under the insulin curve; BMI, body mass index; CANA, canagliflozin; FAI, free androgen index; FBG, fasting blood glucose; FINS, fasting insulin; FSH, follicle-stimulating hormone; HOMA-IR, homeostasis model assessment-insulin resistance; LDL-C, low-density lipoprotein cholesterol; LH, luteinizing hormone; MET, metformin; N/A, not applicable; PCOS, polycystic ovary syndrome; SGLT-2, Sodium-glucose cotransporter-2; SHBG, sex hormone-binding globulin; TG, triglycerides; TC, total cholesterol; TT, total testosterone.
